# Integrated Analysis of a Competing Endogenous RNA Network Reveals a Prognostic Signature in Kidney Renal Papillary Cell Carcinoma

**DOI:** 10.3389/fcell.2020.612924

**Published:** 2020-12-03

**Authors:** Ruyi He, Longyu Wang, Juan Li, Lixin Ma, Fei Wang, Yang Wang

**Affiliations:** ^1^State Key Laboratory of Biocatalysis and Enzyme Engineering, Hubei Collaborative Innovation Center for Green Transformation of Bio-resources, School of Life Sciences, Hubei University, Wuhan, China; ^2^Shine Star (Hubei) Biological Engineering Co., Ltd., Wuhan, China

**Keywords:** KIRP, TCGA, biomarker, prognosis, ceRNA

## Abstract

The kidney renal papillary cell carcinoma (KIRP) is a relatively rare type of renal cell carcinoma (RCC). Currently, most kidney cancer studies primarily focus on RCC, and there has been no investigation to find a robust signature to predict the survival outcome of KIRP patients. In this study, we constructed a competing endogenous RNA (ceRNA) network, including 1,251 lncRNA–miRNA–mRNA interactions. Eight differentially expressed genes (IGF2BP3, PLK1, LINC00200, NCAPG, CENPF, miR-217, GAS6-As1, and LRRC4) based on the TCGA database were selected. The prognostic signature was established by combining the univariate Cox regression method and a stepwise regression method, with its predictive value validated by time-dependent receiver operating characteristic (ROC) curves. In conclusion, we identified eight prognostic signatures with using ceRNA networks. Our study provided a global view and a systematic dissection on KIRP prognosis biomarkers, and the eight identified genes might be used as new and important prognostic factors involved in KIRP pathogenesis.

## Introduction

Kidney cancer has gradually become a commonly diagnosed cancer type worldwide. For years, people diagnosed with kidney cancer had few options for treatment beyond surgery, and survival time rarely exceeded 1 year (Owens, 2016). Kidney renal papillary cell carcinoma (KIRP) is regarded as the second histological type renal cell carcinoma (RCC) with a frequency of about 15–20% (Rhoades Smith and Bilen, 2019). Few investigations have been performed to determine robust signatures for predicting the survival outcomes of KIRP patients with regards to tumor type (Antonelli et al., 2010). According to histologic features, KIRP has been further divided into type 1 and type 2, and type 2 KIRP has been characterized by a high grade, late-stage, and poor prognosis (Pignot et al., 2007). Moreover, type 2 KIRP has more aggressive clinical–pathological characteristics and worse external outcomes (Balakrishnan, 2015). Although molecularly targeted therapies, such as vascular endothelial growth factor (VEGF) receptor and inhibitor and mammalian target of rapamycin (mTOR) inhibitor have been developed for patients, the responses to treatment are varied. However, for patients with metastasis, the benefits provided are modest (Motzer et al., 2014; Engel et al., 2019). Additionally, feasible biomarkers for the prediction of KIRP prognosis and possible new therapeutic targets for KIRP treatment are lacking. Therefore, it is essential to find a robust prognostic predictor for the guidance of clinical therapy of KIRP.

Cancer biomarkers are substances or processes that provide robust prognostic predictors for the risk of developing cancer or measure the risk of cancer progression or potential response to therapy. Long-chain non-coding RNAs (lncRNAs) are seen as the new cancer diagnostic markers and therapeutic targets. They have been observed in oncogenic and tumor-suppressive pathways and are involved in the pathogenesis of cancer and processes, such as unmanageable proliferation or metastasis (Zhu et al., 2013, 2018). Additionally, in the competing endogenous RNA (ceRNA) hypothesis, lncRNAs can bind miRNAs and act as ceRNAs, resulting in the modulation of the mRNA levels targeted by the sponged miRNA (Salmena et al., 2011). Increasing evidence indicates that regulatory networks serve essential roles in the occurrence, development, and regulation of tumors. HAND2-AS1 was shown to function as a competitive RNA that binds with miR-590-3p to influence the expression of potassium sodium-activated channel subfamily T member 2 (KCNT2) (Yu et al., 2020). However, these observations mainly focused on clear cell renal carcinoma, feasible biomarkers for the prediction of KIRP prognosis, and possible new therapeutic targets for KIRP treatment are still lacking. Therefore, a robust signature for KIRP urgently needs to be investigated.

As biomarkers and potential therapeutic targets, ceRNAs have demonstrated their great values for research and clinical applications with regards to tumor pathogenesis. In the present study, we analyzed differentially expressed genes, including mRNA, miRNA, and lncRNA, and constructed a risk score model to predict the overall survival with KIRP patients. Finally, we identified eight biomarkers, including PLK1, IGF2BP3, LINC00200, NCAPG, CENPF, GAS6-AS1, miR-217, and LRRC4, which can serve as predictors for KIRP survival and may become targets for KIRP therapies. This study provides a ceRNA network of KIRP to understand the molecular molecular mechanisms of KIRP progression and provide new targets of the molecular therapy for KIRP.

## Materials and Methods

### Data Preparation and Differentially Expressed Gene Analysis

All primitive data of TCGA-KIRP, including transcriptome data (including RNA-seq and miRNA-seq), and clinical inforsectionmation were acquired from the Genomic Data Commons of the National Cancer Institute^1^. LncRNA and mRNA were commented by EnsDb.Hsapiens.v75 package using R software. In addition, to avoid a low abundance impact on the next procedure, RNAs with TPM-value < 1 and sum(value) < 10 were excluded. The deferentially expressed RNAs (DERNAs; including DEmRNAs and DElncRNAs) and differentially expressed microRNA (DEmiRNAs) were analyzed by DEseq2 package (1.20.0 version) (Love et al., 2014) using R with thresholds of |log2FoldChange| > 2.0 and *p* < 0.01, which were considered statistically different between cancer and normal groups. The heatmap and volcano plot were constructed using the ggplot2 package in R software (Ginestet, 2011).

### Functional Enrichment Analysis

Yu et al. (2012) was used to perform the Gene Ontology (GO) enrichment analysis, including biological process (BP), the cellular component (CC), and molecular function (MF) and Kyoto Encyclopedia of Genes and Genomes (KEGG) pathway analysis. Pathview (Luo and Brouwer, 2013) and enrich plot packages were used to visualize the enrichment results. The cutoff criteria with a value of *p* < 0.05 was considered statistically significant.

### Construction of the ceRNA Network

It is important to match the differentially expressed mRNAs, miRNAs, and lncRNAs according to the ceRNA hypothesis (Thomson and Dinger, 2016). The interactions between miRNAs and mRNAs were evaluated based on the miRTarBase (Release 7.0), and the interactions annotated in this database were supported by strong experimental evidence (reporter assay or western blot). Furthermore, the candidate lncRNA–miRNA interactions were selected based on highly conserved microRNA family data in the miRcode database (miRcode 11). Here, all the miRNAs, lncRNAs, and mRNAs differentially expressed between the tumor and normal tissue. The interactions between DElncRNA-associated DEmiRNAs and DEmRNAs were evaluated to construct the ceRNA network. Cytoscape v3.8 software was used to demonstrate this network visually.

### Protein–Protein Interaction Network Analysis

Protein–protein interaction (PPI) network analysis of the DEmRNAs involved in the ceRNA network were constructed using STRING (version 11.0) with the confidence score > 0.7. The interaction types among proteins were based only on physical interaction and co-expression (Wang et al., 2020).

### Identification of a Prognostic Signature Based on ceRNA Network

The status and survival times of KIRP patients were extracted from the TCGA clinical dataset. Prognostic data were created on the matrix of DEmRNAs, DElncRNAs, and DEmiRNAs identified in ceRNAs. In survival outcomes analysis, patients were classified according to the median expression of RNAs (DElncRNAs, DEmiRNAs, and DEmRNAs) into high or low expression groups. By univariate Cox regression with a logrank test analysis, genes with *p* < 0.01 were selected as primitive biomarkers. Subsequently, we performed stepwise regression to identify the prognostic signatures, and predicted the outcome of the patients with papillary cell carcinoma. The risk score was calculated as the sum of the product of each gene and its coefficient. Patients were divided into high and low-risk groups according to the median of their risk score. Finally, Kaplan–Meier survival curves and receiver operating characteristic (ROC) analysis were applied to validate its accuracy. The 3, 5, and 10 years survival outcomes demonstrated the predictive power of the biomarkers. The expression differences of prognostic signatures in different pathological stages (stage i, stage ii, stage iii, and stage iv) and different tumor types (tumor types 1 and 2) were analyzed in R software.

## Results

### The Identification of Differentially Expressed mRNAs, miRNAs, and lncRNAs

From the TCGA database, a total of 322 raw RNA expression profiles (including 290 cohort KIRP samples and 32 normal samples), 326 raw miRNA-seq data (including 292 cohort KIRP samples and 34 normal samples), and 291 corresponding clinical data of KIRP patients were downloaded. In total, 60,488 transcripts and 1,881 miRNAs were obtained. After selecting the appropriate mRNAs and lncRNA, and removing low abundance RNAs, 18,505 mRNAs, 9,644 lncRNAs, and 869 miRNAs were selected for the differential expression analysis.

**FIGURE 1:**
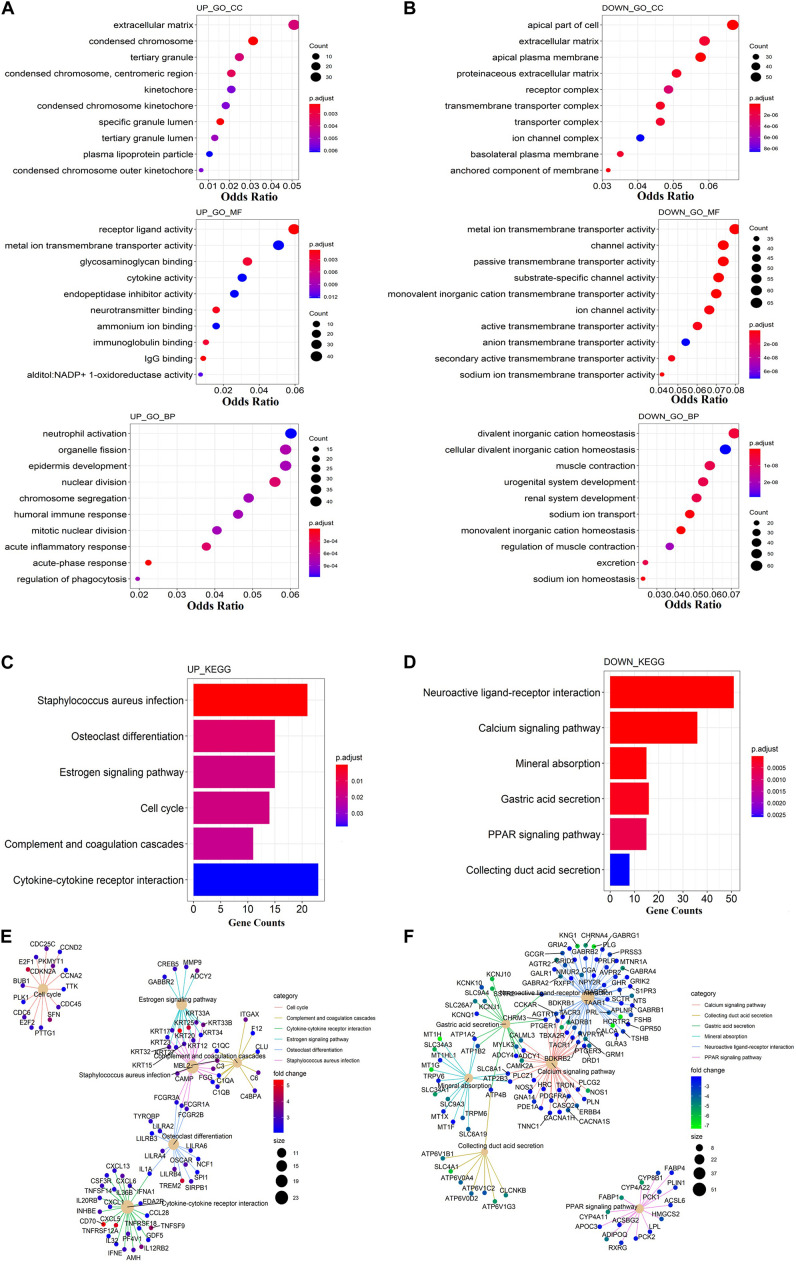
Functional enrichment analysis of DEmRNAs. **(A)** Gene Ontology enrichment analysis of up-regulated mRNAs. **(B)** Gene Ontology enrichment analysis of down-regulated mRNAs. **(C)** KEGG pathway analysis of up-regulated mRNAs. **(D)** KEGG pathway analysis of down-regulated mRNAs. **(E)** The netplot of KEGG pathways means the enrichment of up-regulated mRNAs in different pathways. **(F)** The netplot of KEGG pathways means the enrichment of down-regulated mRNAs in different pathways. The color bar represents the fold change of genes in different pathways. The *y*-axis represents the gene count. The *x*-axis represents the enrichment analysis terms. Each plot’s color represents the *p*-value, while the size represents the gene number in this term.

Finally, compared with normal samples, 1,832 DEmRNA (853 up-regulated and 979 down-regulated, Supplementary Figure 1A),1,036 DElncRNA (458 up-regulated and 578 down-regulated, Supplementary Figure 1B) and 93 DEmiRNA (42 up-regulated and 51 down-regulated, Supplementary Figure 1C) were sorted out with the thresholds of |log2FoldChange| > 2.0 and *p* < 0.01. The heatmap showed the expression tendency of these differential expressed genes in each groups were listed asSupplementary Figures 1D–F).

### Functional Enrichment Analysis of DEmRNAs

To discover the potential biological function of these dysregulated genes, GO and KEGG function enrichment analysis were performed separately on dysregulated mRNAs. Terms with a *p* < 0.05 were considered as statistically significant. In the GO analysis, the up-regulated mRNAs were mainly enriched in the extracellular matrix, receptor–ligand activity, and neutrophil activation (Figure 1A). The down-regulated mRNAs were mainly enriched in the apical part of the cell, metal ion transmembrane transporter activity, and divalent inorganic cation homeostasis (Figure 1B). In KEGG analysis, the up-regulated mRNAs were mainly enriched in staphylococcus aureus infection and cytokine–cytokine receptor interaction (Figure 1C). Meanwhile, the down-regulated mRNAs were mainly enriched in neuroactive ligand–receptor interaction and calcium signaling pathway (Figure 1D). The pathway–pathway interaction network (PPIN) based on the DEmRNAs enriched in the same pathway were constructed. All up-regulated mRNAs were enriched in six pathways. Among which five pathways were connected, including the estrogen signaling pathway, complement, and coagulation cascades, staphylococcus aureus infection, osteoclast differentiation, and cytokine–cytokine receptor interaction (Figure 1E).

The staphylococcus aureus infection pathway is the central pathway that connected all others. Gene FCGR1A, FCGR3A, and FCGR3B were enriched in both the staphylococcus aureus infection and osteoclast differentiation pathways, and belong to the Fc gamma receptor (FcγR), a receptor for the Fc portion of IgG (Bournazos and Ravetch, 2017). Fc gamma receptor is an essential participant in many immune system effector functions, such as opsonized cell phagocytosis, inflammatory mediator release, and antibody-dependent cellular cytotoxicity (Dahan et al., 2015). KRT17, KRT25, KRT33A, KRT33B, KRT20, KRT23, KRT34, KRT12, KRT15, KRT32, and KRT27 were enriched in both the staphylococcus aureus infection and estrogen signaling pathway. These genes encoded for keratins, which could modulate multiple processes including cell migration, tumor growth/metastasis, and development of infections (Toivola et al., 2015). C1Q3, C1QB, and C1QA were enriched in both the staphylococcus aureus infection, complement, and coagulation cascades pathway. These genes encoded for the C1 complement protein, which has anti-cancer effects via immune surveillance and may participate in the aging process (Cho, 2019). All down-regulated mRNAs were enriched in the calcium signaling pathway, collecting duct and secretion, gastric acid secretion, mineral absorption, neuroactive ligand–receptor interaction, and PPAR signaling pathways. Except for the PPAR signaling pathway, the other five pathways are related to each other by some co-expression of genes (Figure 1F).

### Construction of lncRNA–miRNA–mRNA ceRNA Network in KIRP

**FIGURE 2:**
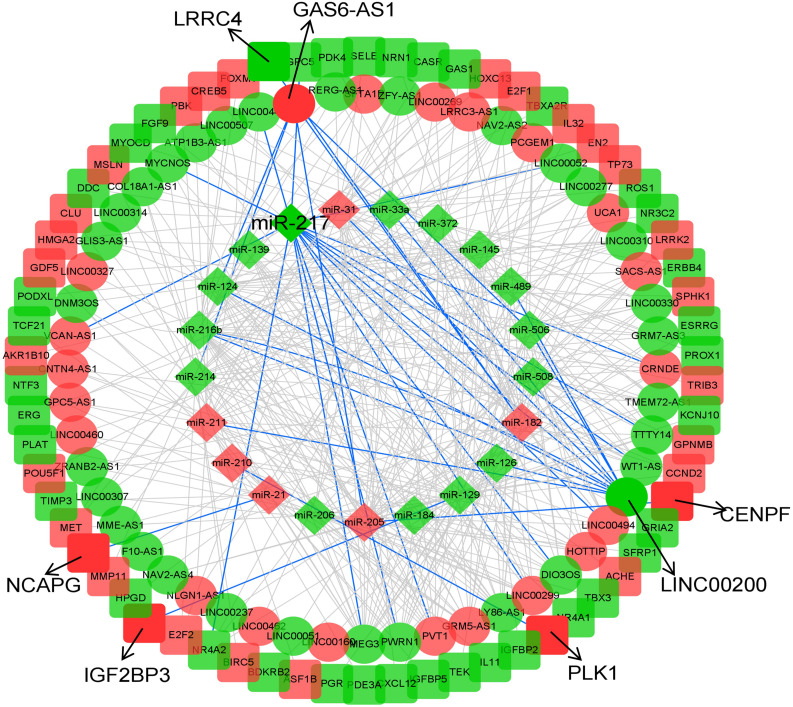
CeRNA network of KIRP. The diamonds indicate miRNAs, oval mean lncRNAs, and square represent mRNAs. Red means up-regulated, and green means down-regulated. The arrow indicates the eight biomarkers of KIRP screened in this study, and the interactions they involved in are shown in blue lines.

To explore the underlying interactions among DEmRNAs, DElncRNAs, and DEmiRNAs in KIRP, a ceRNA network was constructed using Cytoscape v3.8. A total of 1,251 lncRNA–miRNA–mRNA interactions were enrolled in the network, including 21 DEmiRNAs (6 up-regulated, 15 down-regulated), 52 DElncRNAs (21 up-regulated, 31 down-regulated) and 66 DEmRNAs (29 up-regulated, 37 down-regulated) (Figure 2 and Supplementary Table 1). We only selected miRNA–mRNA pairs, whose interactions were supported by strong experimental evidence (reporter assay or western blot) and data from highly conserved microRNA families in the miRcode database (miRcode 11). Detailed information about the expression and association with overall survival outcomes is listed in Supplementary Table 2.

### Protein–Proteome Network Analysis

**FIGURE 3:**
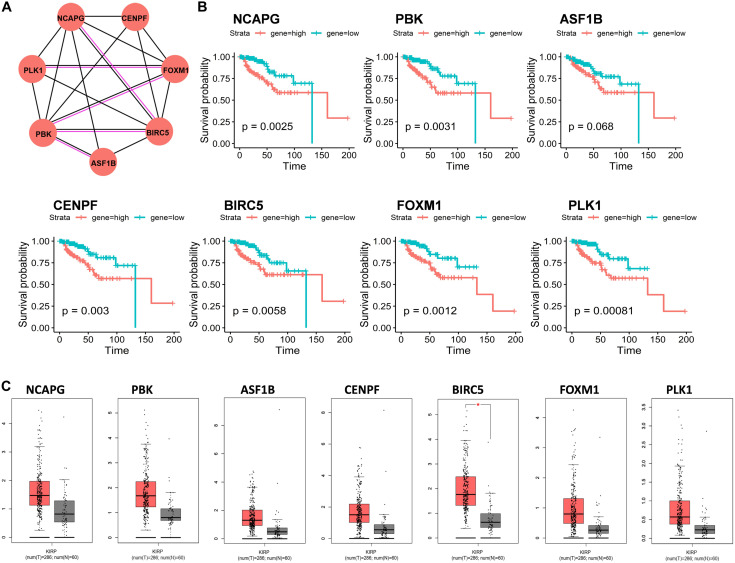
Protein–protein interaction (PPI) network analysis of mRNAs in ceRNA network. **(A)** Seven genes interaction network. Circles indicate the genes in the PPI network, and the connection indicates the potential interaction between different mRNAs. The red line means physical interaction, and the black line means co-expression. **(B)** Overall survival curves of the seven genes in KIRP. *p < 0.05. **(C)** Gene expression of seven hub genes between KIRP tumor and normal tissues.

A total of 66 DEmRNAs in the ceRNA network were used for protein–protein interaction (PPI) network analysis through STRING (version 11.0) with a confidence score > 0.7. The interaction types among proteins were assessed using only physical interaction and co-expression. Finally, seven proteins were selected in PPI, including NCAPG, PBK, ASF1B, CENPF, BIRC5, FOXM1, and PLK1 (Figure 3A). We noticed that seven RNA expression levels were significantly associated with overall survival outcomes except for ASF1B (Figures 3B,C).

### Screen Biomarkers and Construction Risk Model

**FIGURE 4:**
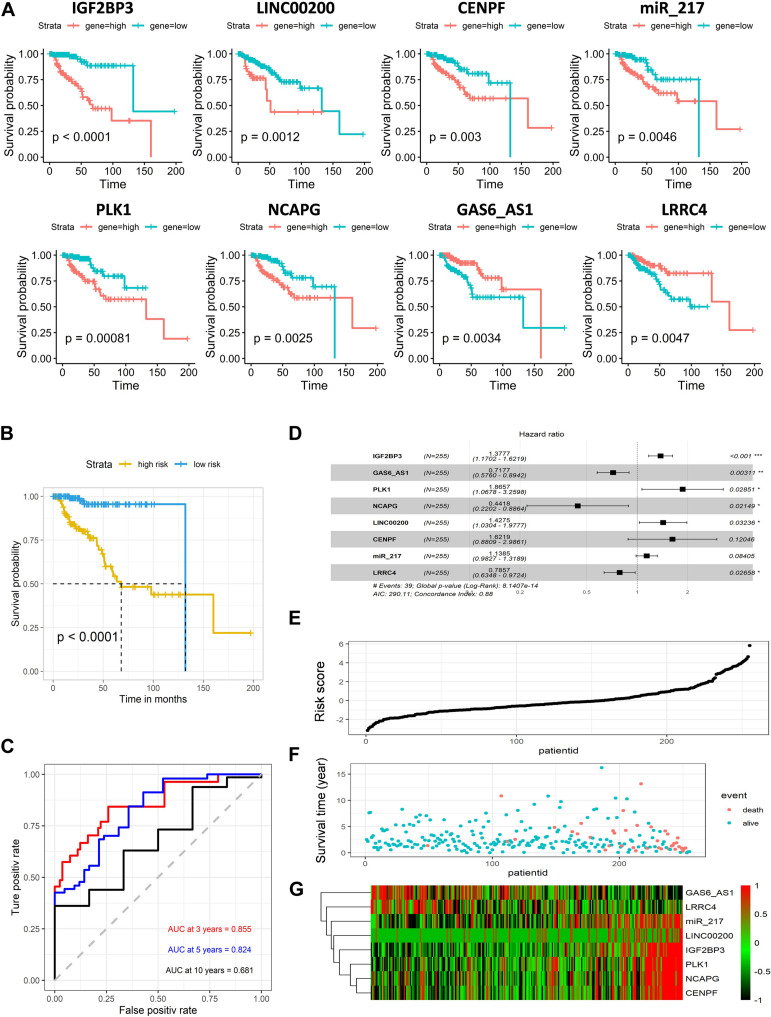
Predictive gene signature analysis. **(A)** Overall survival curves of eight predictive genes in KIRP. **(B)** Kaplan–Meier survival analysis of the risk score for overall survival. Log-rank test was used to compare the survival distribution of these two groups. **(C)** ROC for the prediction of the 3, 5, and 10 years survival based on risk score. Area under the curve is 0.855, 0.824, and 0.681, respectively. **(D)** Forest map based on the risk score model. Left vertical dotted line indicates protective genes and right risk genes. **(E)** Survival status and survival time of each individual. Color of each plot represents the survival status of each patient. **(F)** Risk score of each individual. **(G)** Differentially expressed predictive genes that were enrolled in the risk model heatmap.

All RNAs involved in the ceRNA network were selected for the next step analysis. By univariate Cox regression with a logrank test analysis and a *p* < 0.01, 21 candidates were selected as primitive biomarkers (IGF2BP3, PLK1, IL11, LINC00200, FOXM1, TRIB3, EN2, GPC5-AS1, NCAPG, CENPF, PBK, ROS1, LINC00277, GAS6-AS1, hsa-mir-217, LRRC4, BIRC5, MSLN, HMGA2, SFTA1P, LINC0031). Then a stepwise regression was applied to determine the best signature to predict the outcome of KIRP patients. Eight variables were harvested in the Cox regression model, which had the lowest Akaike information characteristic (AIC). The risk assessment score for the prediction of overall survival was calculated as follows: risk score = 0.320^∗^GF2BP3 + 0.624^∗^PLK1 + 0.356^∗^LINC00200 − 0.817^∗^NCAPG + 0.484^∗^CENPF − 0.332^∗^GAS6_AS1 + 0.130^∗^ miR_217 − 0.241^∗^LRRC4, and the expression of eight genes in KIRP is significantly related to survival (*p* < 0.01) (Figure 4A).

Next, the KIRP patients were divided into a high-risk and low-risk groups using the cutoff value from the median risk score. Kaplan–Meier survival curve analysis demonstrated significant differences in survival time between the high-risk and the low-risk group. The 5 years survival rates for low-risk groups were more than 0.9, and high-risk groups were lower than 0.5 (*p* < 0.001) (Figure 4B). The area under the curve (AUC) in ROC analysis for the 3, 5, and 10 years intervals were 0.855, 0.824, and 0.681, respectively, suggesting that this signature has a high potential for predicting the clinical outcomes of patients with renal papillary cell carcinoma (Figure 4C). The distribution of the risk score, along with the corresponding survival data, demonstrated that the high-risk KIRP patients tended to experience shorter survival times, but low-risk KIRP patients had opposite outcomes (Figure 4D). Overall, the results show that the expression levels of GPC5-AS1 and LRRC4 in the high-risk group were lower, while others were higher, suggesting that the result was consistent with the overall survival analysis (Figures 4E–G).

**FIGURE 5:**
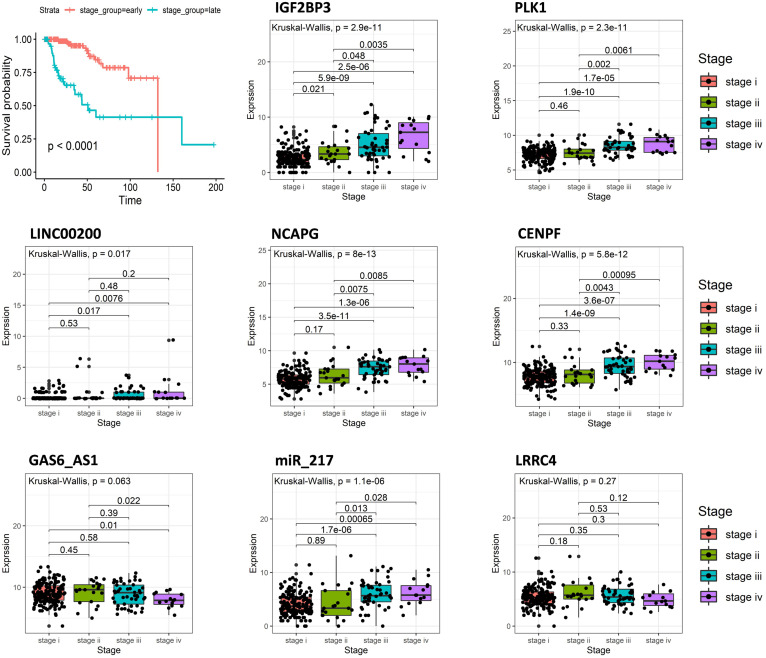
Overall survival curves of tumor stage in KIRP and the expression of eight predictive genes in different tumor stages of KIRP.

Cancer staging and grading are used to predict the clinical behavior of malignancies, establish appropriate therapies, and facilitate the exchange of precise information between clinicians (Telloni, 2017). Overall survival analysis indicated that the tumor stage is closely related to the survival of the KIRP patients. The patients at the early stage (stage i and ii) have longer survival times than late-stage (stage iii and iv) (Figure 5). By analyzing the expression of eight signatures in different tumor stages using R software, it was found that the expression of IGF2BP3, PLK1, LINC00200, NCAPG, CENPF, and miR_217 in the early stage of the tumor were lower than that in the late stage, while GAS6_AS1 and LRRC4 were opposite, and the result was consistent with the above survival analysis.

**FIGURE 6:**
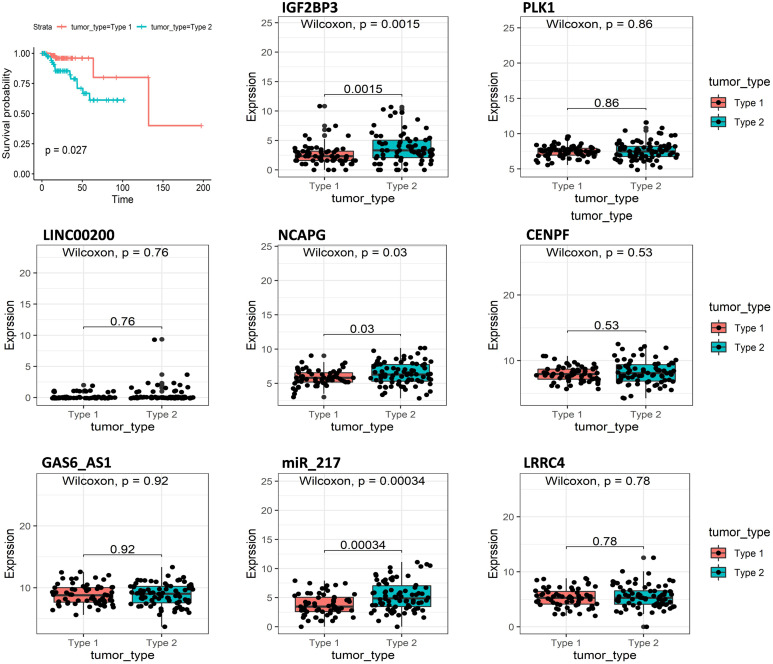
Overall survival curves of tumor type in KIRP and the expression of eight predictive genes in type1 KIRP and type2 KIRP.

Another important indicator of KIRP is the tumor type; type 2 papillary RCC (pRCC) is associated with poorer Eastern Cooperative Oncology Group (ECOG) performance status, higher stage, and grade, and necrosis, leading to worse prognosis compared with type 1 KIRP. Survival analysis indicated that tumor type was closely related to the survival of KIRP patients and that patients with tumor type 2 had shorter survival times compared with tumor type 1 (Figure 6). We analyzed the expression of eight signatures in different tumor types using R software. The results showed that IGF2BP3, NCAPG, miR_217 had significantly higher expression in type 2 KIRP, compared with type 1 KIRP (*p* < 0.05).

## Discussion

Regarding renal cancer, KIRP is generally less well studied than the clear cell type; due to its low incidence, which prevents in-depth investigations of KIRP treatment strategies and prognostic prediction (Wang et al., 2019). In this study, a total of 1,251 lncRNA–miRNA–mRNA interactions were constructed in the ceRNA network, eight genes were identified as prognostic biomarkers in KIRP, including IGF2BP3, PLK1, LINC00200, NCAPG, CENPF, GAS6-AS1, miR217, and LRRC4.

Upon searching for these genes on PubMed, we found that they had been studied for their association with tumor progression as well as their mechanisms, if action with the exception of LINC00200. As an RNA-binding protein (RBP), IGF2BP3 is of particular interest in tumorigenesis and tumor progression because of its over-expression in many tumors and regulation of cell growth and migration, as well as its response to drug (Mancarella and Scotlandi, 2019). Similarly, PLK1 is overexpressed in a large variety of tumors, and this overexpression often confers poor prognosis to the patients (de Carcer, 2019). Furthermore, NCAPG acts as an oncogene in liver hepatocellular carcinoma (LIHC) and plays a role in promoting cell proliferation and anti-apoptosis through the activation of the PI3K/AKT/FOXO4 pathway (Gong et al., 2019). Centromere Protein F (CENPF) also associates with the centromere-kinetochore complex and influences cell proliferation and metastasis in several cancers (Alghamdi et al., 2020). It promotes breast cancer (BC) bone metastasis by activating PI3K-AKT-mTORC1 signaling. CENPF can also be considered as a biomarker of fetal intestinal atresia for prenatal diagnosis (Sun et al., 2019). Many studies reported that miR-217 participated in carcinogenesis and tumor progression in several cancers (Qin et al., 2012). It acts as a tumor suppressor by targeting the oncogene SirT1 in endothelial cells or act as an oncogene by targeting PTEN in kidney cells (Zhang et al., 2016).

The expression of IGF2BP3, PLK1, NCAPG, CENPF, and miR-217 was significantly higher in late-stage (stage i and ii) KIRP compared with the early stage, and overall survival analysis showed that high expression of those genes predicted poor survival and high mortality in KIRP patients. Another prognostic biomarker, GAS6-AS1, is a potential target for therapeutic approaches in hepatocellular carcinoma (HCC), where knock-down of GAS6-AS1 decreased tumor growth *in vivo* (Ai et al., 2020). Similarly, we found that the expression of GAS6-AS1 was also up-regulated in kidney cancer; however, KIRP patients within the high-expression group of GAS6-AS1 had better overall survival compared with the low-expression group.

Analysis of tumor stages showed that the expression levels of GAS6-AS1 decreased in stage IV, which was associated with high mortality. This result indicated that GAS6-AS1 was a critical signature for KIRP and could be used to identify the tumor stage. Consistent with GAS6-AS1, the KIRP patients with high expression of LRRC4 were also associated with higher overall survival. In kidney cancer cells, LRRC4 was under-expressed, and identified as a tumor suppressor gene for gliomas. Moreover, overexpression of LRRC4 suppressed glioma cell growth, angiogenesis, and invasion (Li et al., 2014). Indeed, in our ceRNA network, GAS6-AS1 and LRRC4 both interacted with miR182; thus, we predicted that GAS6-AS1 could bind miR182 and act as a ceRNA, therefore modulating the levels of the LRRC4 in KIRP. Using PubMed, we found that the only report on the function and mechanism of LINC00200, was from Lou et al. (2019). They reported that LINC00200 played a critical part in early stage hepatocellular carcinoma (HCC) after analyzing RNA-sequencing expression data of liver cancer from the TCGA and GEO database (Lou et al., 2019). In our study, high expression of LINC00200 was associated with poor survival outcomes in KIRP patients and higher expression of LINC00200 at later tumor stages, which indicated that LINC00200 may have been an oncogene in renal clear cell carcinoma.

After analyzing the expression of these genes in different tumor types, we observed that IGF2BP3, NCAPG, and miR217 demonstrated higher expression in type 2 compared with type 1 KIRP (*p* < 0.05). The histological characteristics and common mutations were different between type 1 and 2 KIRP. Type 1 KIRP patients have a 5 years survival rate of 95%. In comparison, type 2 papillary RCCs patients are more aggressive, with a 5 years survival rate of 66%. Differing prognoses has prompted the search for a simple, non-invasive means of differentiating type 1 from type 2 KIRP to help guide further management (Young et al., 2017). Differential expression of IGF2BP3, NCAPG, and miR_217 in these two types of KIRP might suggest that these three genes can be used as biomarkers to distinguish the KIRP subtypes, as well as to develop diagnostic methods.

Although this study has some limitations, the findings also provide some direction for future research. The biomarkers identified could be promising therapeutic targets that can lay the groundwork for future experimental research design.

## Conclusion

This work established the disordered ceRNA network of KIRP and identified that IGF2BP3, PLK1, LINC00200, NCAPG, CENPF, GAS6-AS1, and miR217 might be new and important prognostic factors involved in KIRP pathogenesis. The risk score model we developed is helpful in studying the overall survival outcome in KIRP. Additionally, we proposed that GAS6-AS1 can be used as a competitive endogenous RNA for miR-182 to regulate LRRC4 in kidney cells, and IGF2BP3, NCAPG, and miR217 are associated with various types of renal papillary cell carcinoma, which provides us with efficient strategies for subsequent functional studies.

## Data Availability Statement

The original contributions presented in the study are included in the article/Supplementary Material, further inquiries can be directed to the corresponding author/s.

## Author Contributions

YW, FW, and LM conceived and designed the experiments. RH and LW performed the computations and analyzed the data. RH and YW wrote the manuscript. JL wrote and reviewed. All authors read and approved the final manuscript.

## Conflict of Interest

JL was employed by company Shine Star (Hubei) Biological Engineering Co., Ltd. The remaining authors declare that the research was conducted in the absence of any commercial or financial relationships that could be construed as a potential conflict of interest.

## Footnotes

1 http://portal.gdc.cancer.gov

## Supplementary Material

Supplementary Figure 1 Volcano plots of differentially expressed genes in renal papillary cell carcinoma (KIRP) (|log2FoldChange| > 2.0 and adjusted *p* < 0.01) between 289 tumor tissues and 32 normal tissues. The volcano plots described 1,832 DEmRNAs **(A)**, 1,036 DElncRNAs **(B)**, and 93 DEmiRNAs **(C)**. Red stands for up-regulations, blue stands for down-regulations, and gray stands for intermediate in volcanoes. Each point represents a gene. The heatmaps described 1,832 DEmRNAs **(D)**, 1,036 DElncRNAs **(E)**, and 93 DEmiRNAs **(F)**. Orange stands for normal sample, and green stands for a tumor samples.
