# Supplementary figures and images for: Integrated Analysis of a Competing Endogenous RNA Network Reveals a Prognostic Signature in Kidney Renal Papillary Cell Carcinoma
Source: Front Cell Dev Biol. 2020 Dec 3;8:612924. doi: 10.3389/fcell.2020.612924 (PMC7744790; doi:10.3389/fcell.2020.612924)

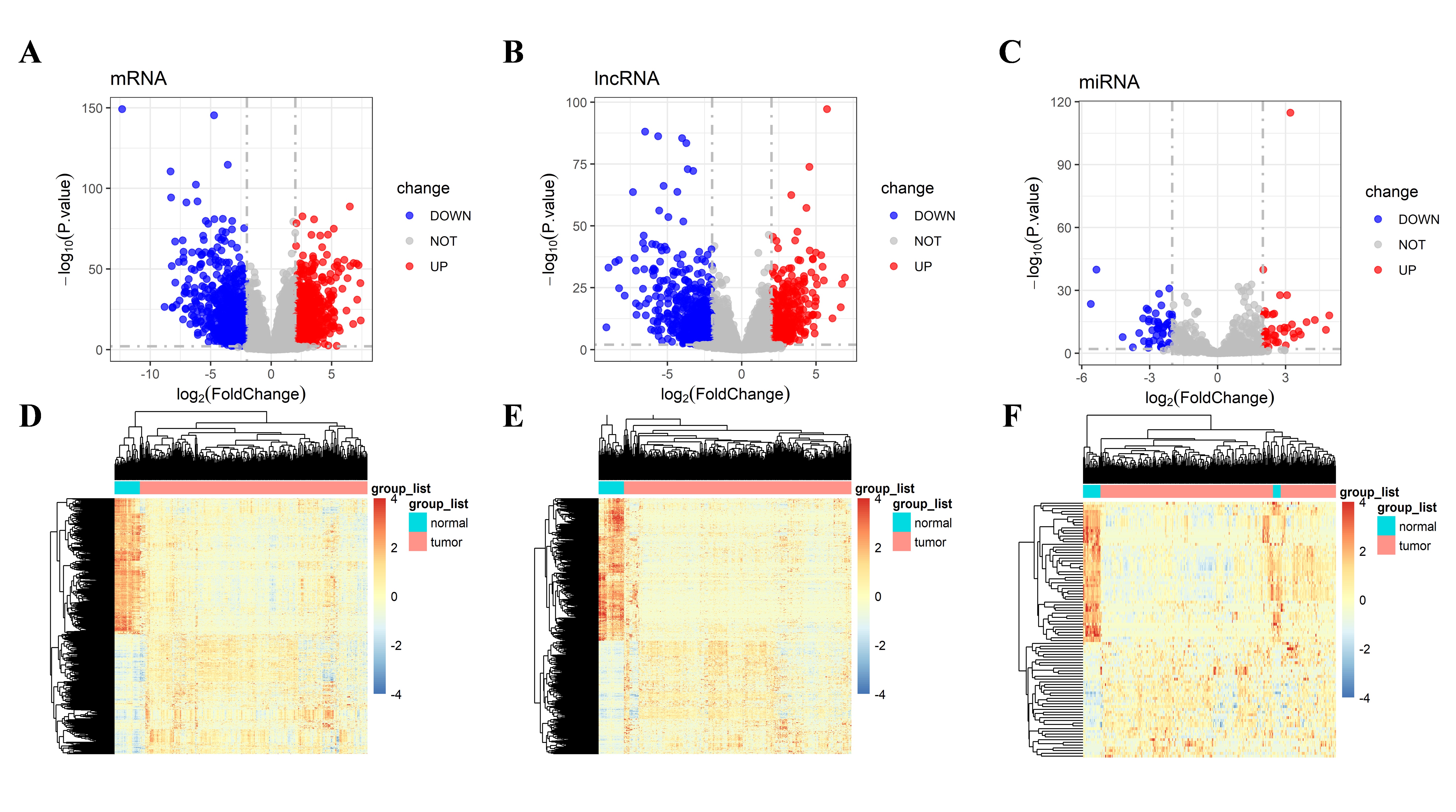

Supplement: Supplementary Figure 1 — Volcano plots of differentially expressed genes in renal papillary cell carcinoma (KIRP) (|log2FoldChange| > 2.0 and adjusted p < 0.01) between 289 tumor tissues and 32 normal tissues. The volcano plots described 1,832 DEmRNAs (A), 1,036 DElncRNAs (B), and 93 DEmiRNAs (C). Red stands for up-regulations, blue stands for down-regulations, and gray stands for intermediate in volcanoes. Each point represents a gene. The heatmaps described 1,832 DEmRNAs (D), 1,036 DElncRNAs (E), and 93 DEmiRNAs (F). Orange stands for normal sample, and green stands for a tumor samples. [file Image_1.jpg]
